# Guideline levels for PFOA and PFOS in drinking water: the role of scientific uncertainty, risk assessment decisions, and social factors

**DOI:** 10.1038/s41370-018-0099-9

**Published:** 2019-01-08

**Authors:** Alissa Cordner, Vanessa Y. De La Rosa, Laurel A. Schaider, Ruthann A. Rudel, Lauren Richter, Phil Brown

**Affiliations:** 1 Department of Sociology, Whitman College, Walla Walla, WA USA; 2 Silent Spring Institute, Newton, MA USA; 3 Department of Sociology and Anthropology, Northeastern University, Boston, MA USA; 4 Department of Health Sciences, Northeastern University, Boston, MA USA

**Keywords:** Drinking water, Emerging contaminants, Exposure assessment, Perfluorinated chemicals, PFAS, Risk assessment

## Abstract

Communities across the U.S. are discovering drinking water contaminated by perfluoroalkyl and polyfluoroalkyl substances (PFAS) and determining appropriate actions. There are currently no federal PFAS drinking water standards despite widespread drinking water contamination, ubiquitous population-level exposure, and toxicological and epidemiological evidence of adverse health effects. Absent federal PFAS standards, multiple U.S. states have developed their own health-based water guideline levels to guide decisions about contaminated site cleanup and drinking water surveillance and treatment. We examined perfluorooctanoic acid (PFOA) and perfluorooctane sulfonate (PFOS) water guideline levels developed by the U.S. Environmental Protection Agency (EPA) and state agencies to protect people drinking the water, and summarized how and why these levels differ. We referenced documents and tables released in June 2018 by the Interstate Technology and Regulatory Council (ITRC) to identify states that have drinking water and groundwater guideline levels for PFOA and/or PFOS that differ from EPA’s health advisories (HAs). We also gathered assessment documents from state websites and contacted state environmental and health agencies to identify and confirm current guidelines. Seven states have developed their own water guideline levels for PFOA and/or PFOS ranging from 13 to 1000 ng/L, compared to EPA’s HA of 70 ng/L for both compounds individually or combined. We find that the development of PFAS guideline levels via exposure and hazard assessment decisions is influenced by multiple scientific, technical, and social factors, including managing scientific uncertainty, technical decisions and capacity, and social, political, and economic influences from involved stakeholders. Assessments by multiple states and academic scientists suggest that EPA’s HA is not sufficiently protective. The ability of states to develop their own guideline levels and standards provides diverse risk assessment approaches as models for other state and federal regulators, while a sufficiently protective, scientifically sound, and enforceable federal standard would provide more consistent protection.

## Introduction

The mobility, persistence, and widespread use of perfluoroalkyl and polyfluoroalkyl substances (PFAS) have resulted in drinking water contamination globally. PFAS were found in the drinking water of more than 16 million Americans in 33 states [1], and a recent analysis indicates that PFAS-contaminated drinking water is much more widespread than previously reported [2]. Surprisingly, despite this widespread contamination [3], ubiquitous exposure [4], and toxicological and epidemiological evidence of health effects [5–7], there are no federal drinking water standards for any PFAS. Instead of a standard, in 2016 the U.S. Environmental Protection Agency (EPA) released a non-enforceable lifetime health advisory (HA) of 70 ng/L for perfluorooctanoic acid (PFOA) and perfluorooctane sulfonate (PFOS), individually or combined. Without an enforceable standard, public water systems (PWSs) are not required to routinely test for PFAS or to treat water exceeding EPA HAs, and so no complete assessment of the prevalence of PFAS in U.S. drinking water exists.

In the absence of federal standards, seven U.S. states have adopted or proposed their own health-based drinking water guideline levels or standards for PFOA and/or PFOS, ranging from 13 to 1000 ng/L. There are important regulatory distinctions between terms such as guidelines, advisories, and standards. For this paper, we use “drinking water guideline levels” as a general term to refer to any risk-based water concentration intended to protect from health effects associated with drinking water consumption, along with more precise terms that are used by individual state or federal agencies, including “health advisory level,” “maximum contaminant level,” or “protective concentration level.” (Tables 1 and 2 use the specific term associated with each agency’s guideline.)

**Table 1:** PFOA drinking water guideline levels

	Advisory level	Critical effect study	Toxicological endpoint	Reference dose	Uncertainty factors	Target population	Water ingestion rate	RSC
U.S. EPA^a^, 2016, Health Advisory Level [35]	70 ng/L	Lau et al. [53]	Developmental	20 ng/kg- day	Total = 300 Intraspecies 10, Interspecies 3, LOAEL to NOAEL 10	Lactating woman	0.054 L/kg-day	20%
Alaska DEC^b^, 2016, Groundwater cleanup level [94]	400 ng/L	Lau et al. [53]	Developmental	20 ng/kg- day	Total = 300 Intraspecies 10, Interspecies 3, LOAEL to NOAEL 10	Child (0–6 years) residential	0.78 L/day, 15 kg body weight (b.w.)	100%
Maine DEP^b^, 2016, Remedial action guideline [95, 96]	130 ng/L	Six studies combined [53, 97–99]	Liver	6 ng/kg-day	Total = 300 Intraspecies 10, Interspecies 3, Database 10	Adult	2 L/day, 70 kg b.w.	60%
Minnesota DOH, 2017, Non-cancer health-based level [42]	35 ng/L	Lau et al. [53]	Developmental	18 ng/kg- day	Total = 300 Intraspecies 10, Interspecies 3, LOAEL to NOAEL 3, Database 3	Infant exposure via breastmilk for 1 year, from mother chronically exposed via drinking water	Derived from internal serum concentrations based on 95% water intake rates and upper percentile breastmilk intake rates	50%
New Jersey DEP, 2017, Maximum contaminant level (recommended) [49]	14 ng/L	Loveless et al. [100]	Liver	2 ng/kg-day	Total = 300 Intraspecies = 10, Interspecies 3, Database 10	Adult	2 L/day, 70 kg b.w.	20%
North Carolina DENR^b^, 2012, Interim maximum allowable concentration (proposed) [58]	1000 ng/L	Butenhoff et al. [101]	Liver	N/A	Total = 30 Intraspecies 10, Interspecies 3	Adult	2 L/day, 70 kg b.w.	20%
Texas CEQ^b^, 2017, Protective concentration level [86]	290 ng/L	Macon et al. [54]	Mammary Gland	15 ng/kg- day	Total = 300 Intraspecies 10, LOAEL to NOAEL 30	Child (0–6 years) residential	0.64 L/day, 15 kg b.w.	100%
Vermont^a^ DEC/DOH, 2016, Primary groundwater enforcement standard [44]	20 ng/L	Lau et al. [53]	Developmental	20 ng/kg- day	Total = 300 Intraspecies 10, Interspecies 3, LOAEL to NOAEL 10	Infant (0–1 year)	0.175 L/kg-day	20%

*Note*: Adapted from ITRC [8]

*CEQ* Commission on Environmental Quality, *DEC* Department of Environmental Conservation, *DENR* Department of Environment and Natural Resources (note that NC DENR is now NC DEQ), *DEP* Department of Environmental Protection, *DEQ* Department of Environmental Quality, *DOH* Department of Health, *RSC* Relative Source Contribution

^a^Applies to PFOA and PFOS individually, as well as the sum of PFOA and PFOS

^b^Alaska, Maine, North Carolina, and Texas follow the EPA’s HA for public and/or private drinking water

**Table 2:** PFOS drinking water guideline levels

	Advisory level	Critical effect study	Toxicological endpoint	Reference dose	Uncertainty factors	Target population	Water ingestion rate	RSC
U.S. EPA^a^ Office of Water, 2016, Health Advisory Level [35]	70 ng/L	Luebker et al. [102]	Reduced pup body weight	20 ng/kg- day	Total = 30 Interspecies 3, Intraspecies 10	Lactating women	0.054 L/kg-day	20%
Alaska DEC^b^, 2016, Groundwater cleanup level [94]	400 ng/L	Luebker et al. [102]	Reduced pup body weight	20 ng/kg- day	Total = 30 Interspecies 10, Intraspecies 3	Child (0–6 years) residential, non- cancer	0.78 L/day, 15 kg b.w.	100%
Maine DEP^b^, 2016, Remedial action guideline [96, 103]	560 ng/L	Seacat et al. [104]	Thyroid effects	80 ng/kg- day	Total = 30 Interspecies 3, Intraspecies 10	Adult	2 L/day, 70 kg b.w.	20%
Minnesota DOH, 2017, non-cancer health-based value [43]	27 ng/L	Luebker et al. [102]	Reduced pup body weight	5.1 ng/kg- day	Total = 100 Interspecies 3, Intraspecies 10, Database 3	Lifetime based on internal serum concentration	Derived from internal serum concentrations based on 95% water intake rates and upper percentile breastmilk intake rates	50%
New Jersey DEP, 2017, Maximum contaminant level, draft [105]	13 ng/L	Dong et al. [106]	Immune response	1.8 ng/kg- day	Total= 30 Interspecies 3, Sensitive subpopulations 10	Adult	2 L/day, 70 kg b.w.	20%
Texas CEQ^b^, 2017, Protective concentration level [86]	560 ng/L	Zeng et al. [107]	Hippocampus synapse structure	20 ng/kg- day	Total = 100 LOAEL to NOAEL 10, Intraspecies 10	Child (0–6 years) residential	0.64 L/day, 15 kg b.w.	100%
Vermont^a^ DEC/DOH, 2016, Primary groundwater enforcement standard [44]	20 ng/L	Luebker et al. [102]	Reduced pup body weight	20 ng/kg- day	Total = 30 Interspecies 3, Intraspecies 10	Infant (0–1 year)	0.175 L/kg-day	20%

*Note*: Adapted from ITRC [8]

*CEQ* Commission on Environmental Quality, *DEC* Department of Environmental Conservation, *DEP* Department of Environmental Protection, *DEQ* Department of Environmental Quality, *DOH* Department of Health, *RSC* Relative Source Contribution

^a^Applies to PFOA and PFOS individually, as well as the sum of PFOA and PFOS

^b^Alaska, Maine, and Texas follow the EPA’s HA for public and/or private drinking water

In this perspective, we compare PFOA and PFOS drinking water guideline levels developed by EPA and seven states, and summarize how and why these levels differ. We aim to provide a useful overview of a rapidly changing regulatory field, identify common factors and decisions that influence guideline development, and examine the importance of social factors. We used tables released by the Interstate Technology and Regulatory Council (ITRC) in June 2018 [8] to identify states with drinking water and groundwater guideline levels for PFOA and/or PFOS that differ from EPA’s HAs. These documents serve as a resource for regulatory personnel addressing PFAS contamination and are updated regularly by a team of environmental professionals. We also contacted state health and environmental agencies to identify and confirm current guideline levels. For all guidelines, we reviewed publicly available risk assessment documents and toxicological summaries prepared by regulatory agencies.

We find that the development of PFOA and PFOS guideline levels is influenced by many scientific, technical, and social factors and decisions including: agency management of scientific uncertainty; an evolving understanding of PFAS health effects; decisions about toxicological endpoints and exposure parameters; and the influence of various stakeholders, including regulated industries and affected communities. We document the rationale used by states to develop guideline levels that differ from those set by EPA. Several states have established guideline levels below EPA’s HA, suggesting that some regulators and scientists view EPA’s approach as not sufficiently protective.

## Perfluoroalkyl and polyfluoroalkyl substances: growing concerns

PFAS as a class include an estimated 4730 human-made and commercially available chemicals, polymers, and mixtures containing chains of fluorinated carbon atoms that are widely used in industrial processes and consumer goods [9]. It is not currently possible to accurately track the use of PFAS individually or as a class in the U.S. because companies can claim production volume data as confidential business information and not disclose it publicly or to EPA. Two PFAS are the most well-known and widely studied. PFOA—previously used to manufacture polytetrafluoroethylene (PTFE) for non-stick coatings such as Teflon™, added as an ingredient in firefighting foams, and created as a byproduct of many other chemical processes—was first used to manufacture commercial products in 1949. U.S. manufacturer DuPont began studying PFOA’s toxicological and exposure concerns starting in the 1960s [10]. PFOS, previously used in fabric protectors such as Scotchgard™, firefighting foam, and semiconductor devices, has been produced since the 1940s. U.S. manufacturer 3M started measuring fluorine levels in blood samples from workers in the 1970s [11]. In 1997, 3M detected PFOS in workers’ blood serum and in samples from U.S. blood banks, intended to represent a control population, and several studies in following years confirmed widespread exposure in the U.S. population [12]. In 2000, 3M announced that it would voluntarily phase out all production of PFOS due to regulatory pressure and concerns over liability [13]. In 2006, following an EPA investigation, eight U.S. chemical manufacturers agreed to phase out all production and use of PFOA and related compounds by 2015 [14]. PFOA and PFOS, both considered long-chain PFAS (perfluorocarboxylic acids with eight or more carbon atoms or perfluorosulfonic acids with six or more carbon atoms [15]), are no longer produced in the U.S., but manufacturing continues in other parts of the world [16] and replacement PFAS are widely used despite growing concerns about persistence, exposure, and toxicity [14, 17–21].

PFAS are important and widespread drinking water contaminants because they are highly persistent, mobile in groundwater, and bioaccumulative [22]. PFAS contamination is often linked to industrial releases, waste disposal and landfill sites, military fire training areas, airports, and other sites where PFAS-containing aqueous film-forming foams (AFFFs) are used to extinguish flammable liquid fuel fires or for firefighter training [1]. Over twenty-five U.S. communities have contaminated water due to releases from manufacturing or industrial waste sites [23], and the Department of Defense (DoD) has identified 401 current or former military sites with known or suspected PFAS contamination, including 126 sites with PFOA or PFOS levels above EPA’s HA, mostly related to AFFF use [24]. In addition to PFOA and PFOS, 57 classes of PFAS have been identified in AFFF and/or AFFF-contaminated groundwater, containing over 240 individual compounds, many of which are poorly characterized in terms of toxicity and environmental fate and transport [25]. Surveillance for PFAS is difficult because of the large number of compounds, many of which lack analytical standards.

Concern about health effects from PFAS is high because of widespread exposure and documented toxicity. Biomonitoring data from the U.S. Centers for Disease Control and Prevention’s National Health and Nutrition Examination Survey (NHANES), a representative sample of U.S. residents, for 12 PFAS from 1999 to 2014 found four PFAS in the serum of nearly all people tested [4, 26]. These PFAS remain widely detected, although population serum levels have generally declined, especially for PFOS, following the phase-outs of U.S. production [26]. An epidemiological study, funded by a DuPont lawsuit settlement, of 69,000 people in the Mid-Ohio Valley who drank water contaminated with at least 50 ng/L of PFOA for at least one year linked PFOA exposure to high cholesterol, ulcerative colitis, thyroid disease, testicular and kidney cancers, and pregnancy-induced hypertension [6]. Other health effects associated with PFOA and several other PFAS based on epidemiological evidence include decreased vaccine response, liver damage, and decreased birth weight [27, 28]. In animal studies, PFAS have shown a variety of toxicological effects including liver toxicity, suppressed immune function, altered mammary gland development, obesity, and cancer [7, 22]. There is concordance between some of the endpoints identified in studies of animals and humans, most notably suppression of the immune system [29]. While there are sufficient data for risk assessment of PFOA, PFOS, and several other PFAS, most PFAS detected in drinking water lack sufficient data for risk characterization [22, 28].

## Drinking water regulation

Public drinking water supplies (PWSs) in the U.S. are regulated under the Safe Drinking Water Act (SDWA), which specifies that EPA is responsible for establishing testing requirements and standards, while states have primary authority to implement and enforce these standards. The SDWA currently regulates over 90 chemical, biological, and radiological contaminants [30]. For most listed contaminants, EPA establishes both a Maximum Contaminant Level Goal (MCLG), a non-enforceable guideline below which no adverse health effects are expected, and a Maximum Contaminant Level (MCL), an enforceable standard for PWSs set as close as feasible to the MCLG while accounting for availability of treatment technologies and cost. PWSs must test for regulated contaminants, which can reveal previously unrecognized contamination, and take any needed action to address violations. Amendments to the SDWA in 1996 removed a requirement for EPA to periodically establish new MCLs and created a more extensive review process, and few additional contaminants have been regulated since 1996 [31]. Private drinking water sources are not regulated under the SDWA. Other laws like the Comprehensive Environmental Response, Compensation, and Liability Act (CERCLA, also known as Superfund) and the Clean Water Act govern groundwater and surface water quality, including responses to contaminated water at industrial sites. States often develop health-based water guidelines to support decisions at these sites, including response to contamination in private wells.

EPA has not set MCLs for any PFAS, though they recently announced their intention to “initiate steps to evaluate the need for a maximum contaminant level (MCL) for PFOA and PFOS” [32]. In an unusual move that reflects the political demand for a federal MCL, 25 U.S. Senators signed a letter urging EPA to develop an MCL for PFAS [33]. Establishment of an MCL would increase EPA’s authority to address PFAS contamination under the Superfund program [33].

The SDWA also requires EPA to consider additional contaminants for regulation. Every five years, EPA must publish a Candidate Contaminant List (CCL) of contaminants being considered for future standards based on health concerns, prevalence in PWSs, and meaningful opportunities for exposure reduction [34]. No MCLs have been developed for contaminants from the CCL since the SDWA 1996 Amendments were enacted [31]. PFOS and PFOA were added to the third CCL in 2009 and were carried forward to the fourth CCL in 2016. To inform this process, every five years EPA must also develop a list of up to 30 contaminants under the Unregulated Contaminant Monitoring Rule (UCMR) program for which PWSs are required to test on a short-term basis to establish their prevalence. In the third cycle (UCMR3; 2013–2015), six PFAS were analyzed by all large PWSs (serving >10,000 customers) and 800 smaller PWSs [3]. EPA decided not to include any PFAS in UCMR4 (2018–2020).

Under the SDWA, EPA can establish HAs for contaminants without MCLs as guidance for federal, state, and local officials. HAs are intended to represent levels of exposure unlikely to cause adverse health effects, considering both cancer and non-cancer endpoints, and can represent specific durations of exposure (one-day, 10-day, or lifetime). Federal HAs and state guidance values can guide response at contaminated sites if drinking water is affected but do not require PWSs to proactively monitor for these contaminants. In 2016, EPA issued HAs for lifetime PFOA and PFOS exposure [3, 35].

Individual states can also establish their own guidelines and regulations, including MCLs, for drinking water contaminants that are not regulated at the federal level, or they can develop stricter guidelines for contaminants with a federal MCL. There is precedent for states to develop drinking water MCLs for contaminants that do not have federal MCLs (e.g., perchlorate in Massachusetts and methyl tertiary-butyl ether in California) or to develop MCLs that are more stringent than EPA's (e.g., several volatile solvents in New Jersey and California) [36–38]. These state standards and guidelines may apply to PWSs or be used as screening or cleanup levels at contaminated sites (e.g., sites with contaminated groundwater or drinking water). However, some states are precluded by state law from developing their own guidelines or standards, and other states may lack the resources to do so. For instance, Pennsylvania identified lack of funding, technical expertize, and occurrence data as challenges in setting a state standard for PFOA and PFOS [39].

## Variation in PFOA and PFOS drinking water guideline levels

**Fig. 1:**
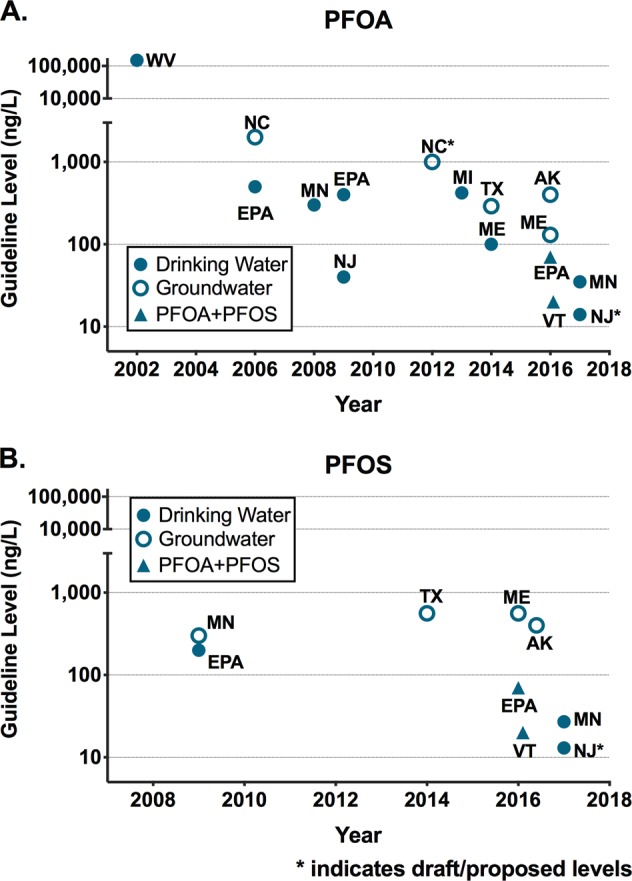
Timeline of Select PFOA and PFOS Drinking Water Guideline Levels. (**a**) PFOA and (**b**) PFOS water guideline levels have decreased over time. Several states have developed guidelines for PFOA or PFOS individually (circles), while Vermont (VT) and EPA have guidelines that apply to PFOA and PFOS individually or combined (triangles). PFOA and PFOS water guidelines can apply to different water types such as public drinking water (closed circles) or groundwater, e.g., at contaminated sites (open circles)

In the absence of federal MCLs, multiple states have proposed or adopted drinking water guidelines or standards for PFOA and/or PFOS (Fig. 1). The first PFOA guideline level of 150,000 ng/L was developed in West Virginia in 2002 in response to PFOA-contaminated drinking water near a DuPont facility. In 2006, EPA issued a screening level of 500 ng/L for PFOA for West Virginia sites contaminated by DuPont [40]. In 2009, EPA developed provisional, short-term HAs of 400 ng/L for PFOA and 200 ng/L for PFOS in response to a contaminated site in Alabama. Around the same time, states such as Minnesota and New Jersey developed PFOA guidelines and standards that were lower than the EPA’s short-term HA. In February 2016, in response to concerns about water contamination near an industrial facility, Vermont drafted a drinking water HA for PFOA of 20 ng/L that was finalized in March 2016 [41]. In May 2016, EPA finalized its lifetime HA of 70 ng/L for PFOA and PFOS individually or combined [3, 35]. Shortly after, Minnesota, building off the EPA’s 2016 risk assessments, developed state guideline levels of 35 ng/L PFOA and 27 ng/L PFOS that were lower than the EPA HAs [42, 43], and Vermont referenced the EPA’s risk assessment in revising its HA to 20 ng/L for PFOA and PFOS individually or combined [44]. In 2017, New Jersey recommended MCLs of 14 ng/L for PFOA and 13 ng/L for PFOS, which, if adopted, would be the first standards to require surveillance by PWSs for PFOA and PFOS, as well as being the lowest guideline levels in the U.S.

We analyzed fifteen current or proposed water guidelines or standards for PFOA or PFOS that are the most recent guidelines for the EPA and each state: EPA’s PFOA and PFOS HAs, seven state guidelines for PFOA, and six state guidelines for PFOS (Tables 1 and 2). Some states (e.g., New Jersey and North Carolina) have older adopted guidelines, as well as newer proposed guidelines that have not yet been formally adopted; in these cases, we analyzed the more recent, proposed guidelines. Some guideline levels apply to individual chemicals, while others are based on the sum of multiple PFAS. For example, the EPA HA applies to PFOA and PFOS combined, and the Connecticut, Massachusetts, and Vermont guidelines refer to the sum of PFOA, PFOS, and three other PFAS [45–47]. Eight states (Colorado, Delaware, Massachusetts, Minnesota, New Jersey, North Carolina, and Texas) have developed guideline levels for PFAS other than PFOA and PFOS. Many other states follow EPA’s 70 ng/L HA level and are not included in our analysis or shown in the Figure or Tables.

The most recent proposed state guideline levels for PFOA vary by a factor of 70, from 14 ng/L (New Jersey) to 1000 ng/L (North Carolina; Table 1). For PFOS, the seven guidelines vary by a factor of 43, from 13 ng/L (New Jersey) to 560 ng/L (Maine and Texas; Table 2). Alaska, Maine, and Texas follow EPA’s HA for public and/or private drinking water supplies but have developed higher guideline levels for other contaminated water and site remediation intended to be protective of drinking water exposures from groundwater at those contaminated sites.

## PFOA and PFOS health-based risk assessment

Comparing the risk assessments developed by states and EPA to derive these guideline levels highlights the scientific uncertainty and assumptions that underlie these decisions. Tables 1 and 2 summarize critical components of each assessment: toxicological endpoint, critical study, uncertainty factors, target population, and exposure parameters.

### Toxicological and dose-response assessments

Risk assessment is used to develop health-based guideline levels. Scientists first review toxicological, epidemiological, and mode of action studies to identify the critical effect, the most sensitive adverse endpoint that is considered relevant to humans. Four of the eight guideline levels for PFOA are based on developmental effects, three are based on liver toxicity, and one is based on mammary gland development effects. Of the seven guideline levels for PFOS, four are based on reduced pup body weight, one is based on thyroid effects, one is based on suppressed immune response, and one is based on developmental neurotoxicity. New Jersey’s recommended PFOS MCL, the lowest in the country, is the only assessment to use immune response as the critical endpoint.

The critical effect serves as the starting point for deriving a point of departure (POD), the point on the dose-response curve to which uncertainty factors (UFs) are applied, such as a No Observed Adverse Effect Level (NOAEL) or Lowest Observed Adverse Effect Level (LOAEL). In PFAS assessments, toxicokinetic adjustments were made to account for slower excretion of PFOA and PFOS in humans compared to animals, either by calculating a Human Equivalent Dose based on doses used in animal studies (most states and EPA) or by converting serum levels based on animal studies into serum levels in humans (New Jersey). This is a particularly important consideration for PFAS because of substantial variation in PFAS toxicokinetics among humans and test animals [48]. There are also sex-specific and species-specific differences in the excretion rates of PFAS. For example, PFOA has a very short half-life in female rats (4–6 h) due to rapid excretion [48], which makes the female rat a poor model for studying chronic or developmental effects of PFOA exposure since it is unlikely to reach a steady-state level when administered on a daily basis.

After a POD is derived, UFs are applied to the POD for non-cancer endpoints to estimate a reference dose (RfD), the daily dose expected to be without harm. PFOA and PFOS assessments utilized various UFs to account for: potential differences in sensitivity among people (intraspecies UF) and between humans and animals (interspecies UF); gaps in toxicity data (database UF); and critical effect studies for which the POD was a LOAEL (LOAEL-to-NOAEL UF). UFs were applied differently across PFOA and PFOS assessments. The EPA and all state-based PFOA assessments except for North Carolina have total UFs of 300. North Carolina, the state with the highest proposed PFOA guideline level, has a total UF of only 30 based on intraspecies and interspecies UFs. For PFOS, Texas and Minnesota have total UFs of 100 while other states and the EPA have total UFs of 30. Texas includes a UF for LOAEL-to-NOAEL extrapolation, and Minnesota a database UF to account for potentially more sensitive immune effects.

States and EPA developed guideline levels that are based on a single critical effect but are intended to also be protective of other cancer and non-cancer health outcomes. Though New Jersey’s recommended PFOA MCL is based on an RfD for liver toxicity, the state also considered whether the MCL would be protective for cancer endpoints or mammary gland development. Their assessment based on increased incidence of testicular tumors in rats arrived at the same 14 ng/L guideline level [49]. Their assessment based on altered mammary gland development produced a recommended PFOA MCL equivalent to 0.77 ng/L—18 times lower than the RfD used to derive the proposed MCL. This lower MCL was not recommended due to the lack of precedent for mammary gland development as a critical endpoint in risk assessment, although an additional UF of 10 for sensitive effects was applied to protect for this endpoint [49]. Vermont and EPA both calculated PFOA guideline levels for testicular cancer and determined that guideline levels based on the non-cancer endpoints were more protective. Minnesota did not derive a cancer-based PFOA guideline level, instead concluding that existing data were inadequate for assessing carcinogenic potential and that the non-cancer guideline was protective of potential cancer effects. All PFOS guideline levels are based on non-cancer endpoints, with most assessments indicating that cancer endpoints were reviewed and found to be not sufficiently well-studied to establish a cancer-based guideline level.

### Exposure assessment

Following the derivation of an RfD, exposure assumptions are used to establish a concentration in drinking water that is intended to be health protective, usually targeted to protect sensitive subgroups such as children. Exposure assessment relies on assumptions about the target population, water ingestion rates, and proportion of the daily dose supplied by drinking water relative to other exposure sources, known as the relative source contribution (RSC). These assumptions may vary based on the type of guideline (e.g., groundwater or drinking water).

In PFOA and PFOS assessments, target populations to be protected differed across states, even among those that used the same critical endpoint and/or had a similar RfD. EPA, Alaska, and Vermont derived the same critical endpoint and RfD for PFOA, yet their guideline levels ranged from 20 ng/L (Vermont) to 400 ng/L (Alaska), a 20-fold difference, because they used different exposure parameters. Vermont and EPA selected different target populations (infants for Vermont, lactating women for EPA), leading to divergent water ingestion rates and consequently different PFOA guideline levels for water. Minnesota’s assessment is based on exposure for breastfed and formula-fed infants. Texas assumed that children’s water consumption is 0.64 L/day, while Alaska assumed it is 0.78 L/day.

States also differed in their selection of RSC values. Most states and EPA assumed an RSC value of 20% for drinking water, which limits daily exposure from contaminated drinking water to 20% of the RfD so that additional exposures from other sources, such as consumer products or diet, do not push total exposure above the RfD. All other exposure assumptions being equal, lower RSC values correspond to lower drinking water guideline levels. Minnesota and Maine used human biomonitoring studies to derive RSCs for PFOA and PFOS ranging from 20% to 60%. Alaska and Texas used a 100% RSC, meaning that for people drinking water at their guideline, any dietary and consumer product exposures would raise their intake above the RfD. The Alaska and Texas PFOA and PFOS guidelines, which are 4–8 times higher than EPA’s HAs, were developed for remediation and clean-up of contaminated sites, and these states use EPA’s HAs as limits for PWS drinking water.

## Factors contributing to variation in PFAS guideline levels

Considering the most recent adopted or proposed PFOA and PFOS water guideline levels at the federal and state levels, the range of “safe” levels in drinking water spans almost two full orders of magnitude, from 13 to 1000 ng/L. This variation reflects responses to scientific uncertainty in risk assessment, technical decisions and capacity, and social, political, and economic influences from involved stakeholders.

### Scientific decisions

Differences between water guidelines in part reflect responses to scientific uncertainty. As described above, health risk assessment requires many assumptions and estimates in order to predict a safe exposure for humans. These include identifying critical effects, addressing inter-species and intra-species variation, quantifying other uncertainties, and selecting exposure parameters. Many areas of toxicity and exposure research on PFAS have not achieved scientific consensus so risk assessors make diverse choices.

Another important consideration in these and future assessments is the consideration of epidemiological evidence. Many of the assessments noted that effects in human studies were consistent with the critical effect in animal studies, giving greater confidence to the assessment. However, all of the assessments used dose-response data from animal studies as a basis for their drinking water levels. New Jersey assessments compared their target PFOS serum level of 23 ng/mL with the midrange of serum levels in epidemiological studies that reported effects (6–27 ng/mL) and with U.S. serum levels (median 5 ng/mL, 95%ile 19 ng/mL, from 2013–2014 NHANES) [50]. Based on this comparison, New Jersey recognized the need to minimize any additional exposures from drinking water since the population is already approaching effect levels from the epidemiological studies and risk-based exposure limits. While risk assessors generally expect their approaches to produce exposure levels that will be protective for exposed humans, PFOS immune effects in children are reported at lower exposures than the EPA’s drinking water advisory levels [50]. A recent assessment used epidemiological data to propose a drinking water guideline of 1 ng/L to prevent additional increases in serum PFOS levels [51]. Several other endocrine disrupting compounds show effects in humans at exposures below EPA risk-based exposure limits, including di-(2-ethylhexyl)phthalate (DEHP) and polybrominated diphenyl ethers (PBDEs) [52].

The number of peer-reviewed scientific articles on PFAS has increased dramatically since 2000, while federal and state PFAS drinking water guideline levels have generally decreased over this time (Fig. 1). This demonstrates a common phenomenon: initial risk assessments based on limited data are often shown not to be health protective once more complete data become available. For PFOA and PFOS, the tightening of the guidelines is largely not due to new toxicology studies, but rather to improved exposure research, advances in analytical measurement technologies, improved biomonitoring and toxicokinetic data, and epidemiological findings. For example, both of EPA’s PFOA HAs, the 2009 provisional HA for short term exposure and the 2016 lifetime HA for chronic exposure, are based on developmental effects from the same mouse study [53], but different exposure parameters and toxicokinetic assumptions led to a much lower HA in 2016. Seven of the eight PFOA assessments, all released between 2012 and 2017, use critical endpoints from studies published in 2006 or earlier. EPA’s assessments are also influential: once EPA derived RfDs for the 2016 HAs, states such as Minnesota and Vermont used these RfDs along with different decisions about exposure parameters, resulting in lower guideline levels.

The most sensitive toxicological endpoints—altered mammary gland development and suppressed immune function—were not the basis for EPA’s PFOA and PFOS HAs. However two states, Texas and New Jersey, did use these endpoints as the basis for their PFOA Protective Concentration Level (PCL) and PFOS MCL, respectively. Although in utero PFOA exposure has been shown to alter mammary gland development in rodents [54, 55], this specialized endpoint is not routinely evaluated in regulatory toxicity studies and there is limited precedent for using it in risk assessment [56, 57]. To the best of our knowledge, altered mammary gland development has never been used as a critical endpoint for the basis of any federal regulatory risk assessment in the United States.

Texas based their PFOA PCL on altered mammary gland development from a full gestational study in mice since this endpoint showed a dose response. Texas determined this RfD to be protective of increased liver weight effects observed in several other studies. New Jersey’s PFOA assessment did not use mammary gland changes as the critical effect but did recognize that it was most sensitive and included an additional UF for database uncertainty related to mammary gland effects. Minnesota identified delayed mammary gland development as a co-critical effect, but did not include additional UFs. North Carolina and EPA cited uncertainty related to variation in response between mouse strains, inconsistent methods across studies, and questions about toxicokinetics as challenges for using this endpoint [35, 58], though risk assessments commonly rely on endpoints for which there is substantial intra- and inter-species variation in sensitivity. Most notably, EPA discounted effects on mammary gland development because these alterations were not associated with decreased lactation function and the mode of action for mammary gland development effects is not well described. Though EPA was reluctant to consider the changes adverse, a substantial body of scientific work suggests that altered mammary gland development is likely to influence later breast cancer risk [57]. New research to better characterize these associations is important because many endocrine disruptors alter mammary gland development if exposure occurs in utero or early in life. Routine assessment of mammary gland development in toxicity studies of endocrine disruptors will be informative and improve understanding of these changes and reduce uncertainty for future risk assessments.

New Jersey used decreased plaque forming cell response (suppressed immune function) as the basis for their PFOS MCL, noting also the consistency between this effect and decreased vaccination response in epidemiological studies. Minnesota identified suppressed immune function as a co-critical effect and included a database UF of 3 for immunotoxicity. While the EPA indicated a concern for adverse immune effects, it chose not to use suppressed immune function as the basis for the PFOS HA because a “lack of human dosing information and lack of low-dose confirmation of effects in animals for the short-duration study precludes the use of these immunotoxicity data in setting the RfD” [35]. The New Jersey assessment includes a rebuttal of EPA’s decision, noting that EPA has used this endpoint as a basis for RfDs for other chemicals [50].

### Social, political, and economic influences

While risk assessments such as these PFAS water guidelines are presented as being based solely on scientific considerations, this process is also influenced by political, social, and economic factors [59–63]. For PFAS, much like other high-value products such as tobacco, the landscape of what is scientifically known and unknown about their health and environmental impacts is influenced by the context of knowledge production. Internal industry documents reveal a broad “science-based defense strategy” to “command the science” on PFAS, ranging from suspected influence on state environmental protection agencies in the case of West Virginia, to the selective peer review publication of internal research, to paying academic scientists to influence the peer-review process [10, 64, 65].

PFAS manufacturing companies have influenced PFAS water guidelines in both overt and subtle ways. For example, in 2001 EPA and West Virginia Department of Environmental Protection (WVDEP) learned that DuPont scientists had found high levels of PFOA in regional drinking water. The following year, DuPont collaborated with WVDEP and a state-appointed C8 Assessment Toxicity Team to develop a screening level of 150,000 ng/L, despite numerous conflicts of interest and DuPont’s own internal guideline of 1000 ng/L [10, 66].

Economically invested corporations have indirectly influenced the development of PFAS drinking water guideline levels through the strategic production and dissemination of industry-friendly research, a well-documented pattern in environmental health [67]. Recent litigation by the State of Minnesota Attorney General against 3M revealed internal correspondence between the company and academic scientists paid as consultants. In one instance, an academic scientist hired by 3M wrote in private emails that he intentionally described his work reviewing articles for publication as “literature reviews” in order to avoid a paper trail to 3M, bragged about rejecting an article on PFAS health effects, and offered to pass unpublished articles to peer reviewers recommended by 3M, clear violations of scientific norms [64].

Industry sponsorship of toxicological research and risk assessments can also influence the developments of guidelines through the “funding effect” in which funding source influences published outcomes [68–70]. Studies or assessments funded by a company or industry that benefits financially from the product under investigation are less likely to identify risks and more likely to demonstrate efficacy (or ambiguity), while the opposite is true of studies funded by government agencies or independent parties. Of the eight critical studies used to derive PFOA (*n* = 5) or PFOS (*n* = 3) guidelines, five were conducted by PFAS manufacturers (3M or DuPont), two were conducted by the U.S. government (EPA or NIEHS), and one was conducted by academic researchers with funding from the Chinese government. North Carolina’s PFOA guideline, the highest in the country, heavily references a risk assessment conducted by industry consultants [71]. However, the small number of PFAS guidelines prevents any quantitative analysis of funding effects. Risk assessments, which rely on many assumptions to estimate human exposure and toxicity in the absence of data, are more vulnerable to funding effects. For example, a 2009 PFOA risk assessment funded by DuPont and 3M identified 880 ng/L as “a reliable, albeit conservative” level for an MCL, over 12 times higher than the EPA HA [71].

Industry-funded research may also influence the overall landscape of PFAS research because it is selectively produced and shared [10]. For example, most research conducted by chemical companies is never published or made public, even when disclosure could be useful for assessing chemical risk. Major PFAS manufacturers have repeatedly violated information disclosure requirements under the Toxic Substances Control Act (TSCA) Section 8(e) by not disclosing information on substantial risks related to PFAS in production [72, 73]. This practice has resulted in multi-million dollar fines and also delayed the production of science on environmental and human health effects of PFAS by decades [74, 75]. Today, PFAS manufacturers commonly assert that information on production quantities, use in consumer goods, and chemical identity is confidential business information, creating barriers for scientists and regulators seeking to prevent harmful exposures.

Unlike some states where limited regulatory appetite and strong industry and political influence may slow progress on protecting public health by establishing PFAS water exposure limits, other states have developed scientifically sound PFAS guideline levels in response to discoveries of local contamination. For example, after the discovery of PFOA contamination in Hoosick Falls, New York, a resident in nearby North Bennington, Vermont raised concerns to local legislators. The state of Vermont reacted quickly, first creating a PFOA HA of 20 ng/L and then using that HA to develop a groundwater enforcement standard. Testing of private wells by Vermont’s Department of Environmental Conservation found PFOA concentrations well above the state’s HA, prompting the state to quickly provide bottled water and conduct additional water testing, soil sampling, and blood testing of local residents [41, 76, 77]. In contrast, North Carolina, home to a major Chemours PFAS manufacturing facility, has not updated their PFOA interim maximum allowable concentration of 2000 ng/L, the highest in the United States, despite a 2012 proposal that this guideline be lowered to 1000 ng/L. North Carolina recently developed the nation’s first drinking water provisional health goal for GenX (hexafluoropropylene oxide dimer acid), a PFOA replacement, following discovery of widespread contamination in local rivers that are used for drinking water [78]. This example demonstrates that local pollution concerns can motivate states to develop guidelines or standards without waiting for federal precedent. Legislators at the state and federal level may play an increasing role going forward. Recent examples include a legislatively proposed 5 ng/L level for PFOA and PFOS in Michigan and pressure from 25 U.S. Senators on EPA to develop a PFAS MCL [33, 79].

## Discussion and conclusion

The wide range of PFOA and PFOS guidelines—up to 70-fold difference between states—as well as the lack of enforceable MCLs and deference by many states to EPA’s HA of 70 ng/L have significant public health implications. Our finding that some states have taken additional steps beyond federal action in evaluating and/or regulating PFAS is consistent with states taking more health-protective action on other chemicals, including flame retardants and bisphenol A [80, 81].

EPA’s HAs do not require ongoing monitoring by PWSs or treatment of water that exceeds the HAs, though in practice many other entities use the HA to make remediation decisions. If MCLs existed for PFAS, regulators would have greater authority to take action at contaminated sites under CERCLA, and DoD sites would be able to move forward with remediation of contaminated sites [33]. In addition, given the toxicity, persistence, and mobility of PFAS, systematic screening of PWSs is a logical approach to protect public health. Some states, including Michigan and Washington, are testing PWSs for certain PFAS [82, 83], and New Jersey’s recommended MCLs would require routine testing. In the absence of MCLs, guidelines are applied only after contamination is discovered by other mechanisms, for example, when residents seek water testing near known industrial sites. Public and regulatory awareness of PFAS water contamination has benefited from nationwide testing initiatives, including EPA’s UCMR testing and DoD identification of PFAS-contaminated military sites. The recently authorized nationwide study on PFAS exposure at military sites may be particularly useful in raising awareness and potentially supporting further regulatory action [84].

Regulatory and scientific attention to PFAS has focused on PFOA and PFOS, but the scope of potential PFAS contamination is much broader. While there are data available to support risk assessment for several additional PFAS, including perfluorobutyrate (PFBA), perfluorobutanesulfonic acid (PFBS), perfluorononanoic acid (PFNA), perfluorohexane sulfonic acid (PFHxS), and GenX, there are no studies on prevalence, exposure, and toxicity for many other PFAS, or even analytical methods to detect them [22]. PFAS as a class are generally persistent and mobile, and the few that have been adequately tested share some toxic effects and exposure characteristics with PFOA and PFOS [14, 18–21, 85]. The lack of information and potential scope of the contamination poses significant challenges for protecting public health. The fact that several guideline levels, including EPA’s HAs, apply to the total concentration of multiple PFAS suggests that regulatory agencies are attentive to PFAS as a class, not just as individual compounds. In the absence of toxicity data on individual chemicals, regulators could use well-characterized PFAS as analogues for deriving RfDs and guideline levels, or could develop methods to regulate PFAS as a class, although this would involve additional assumptions and uncertainties. Texas developed PCLs for 16 PFAS, deriving RfD values for PFAS with limited toxicity data using well-characterized PFAS as surrogates [86]. Relative potency estimates have been used in other chemical classes, such as polycyclic aromatic hydrocarbons and dioxins, and are being explored for PFAS [87]. Some existing regulations treat all long-chain PFAS similarly. The U.S. Food and Drug Administration (FDA) has restricted all long-chain PFAS as a class [59, 88], and EPA’s PFOA Stewardship Program includes PFOA and all “precursor chemicals that can break down to PFOA, and related higher homologue chemicals” [89]. The similarities between many PFAS in terms of chemical structure and exposure potential, combined with potential differences in toxicity and the long time required to gather sufficient data, further raise the importance of limiting manufacture and use of PFAS before they become exposure concerns.

EPA-validated drinking water testing protocols exist for 18 PFAS (EPA Method 537), though validated methods are lacking for other PFAS and other media, such as groundwater. It is difficult to understand why EPA has not included any  PFAS in the fourth cycle of UCMR testing, despite significant data gaps regarding the extent of drinking water contamination with other PFAS and the need for surveillance using lower detection limits [90]. The focus of current water screening and treatment efforts solely on removing PFOA and PFOS is concerning because carbon filtration designed to remove long-chain PFAS is less effective at removing short-chain PFAS and PFAS transformation products likely present in AFFF-contaminated water [91] and at PFAS production sites [21].

Our review of PFAS drinking water guideline levels highlights opportunities to extend risk assessment methods to include some important endpoints such as mammary gland development and immune function. Reports of immunosuppression in children with exposures within the exposure range prevalent in the general population have raised concern that EPA’s HAs are not adequately protective, since modeling indicates that consumption of drinking water at 70 ng/L would substantially increase PFOA and PFOS blood levels above current U.S. background levels [51]. Additionally, New Jersey’s PFOA assessment estimated that the RfD for mammary gland changes is below median blood levels in the general population [49]. Grandjean and Clapp [51] proposed that a drinking water concentration of 1 ng/L for PFOA and PFOS would not be expected to lead to an increase in population-level blood serum levels above current U.S. averages.

Our analysis also highlights opportunities to consider epidemiological data more carefully in conjunction with toxicological and exposure data. Despite a relatively robust epidemiological literature for PFOA and PFOS, only New Jersey showed how their target blood level was in the range of exposures in human studies that show effect on vaccine response. New Jersey also used human biomonitoring data to illustrate that even small increases in exposure are problematic because current exposure levels are near levels associated with health effects [22]. However, the environmental co-occurrence of multiple PFAS is a challenge for using epidemiological data to develop guideline levels for individual PFAS [92]. Considering information from human biomonitoring and epidemiology adds important context to the risk assessment process.

The scientific and regulatory landscape on PFAS continues to evolve rapidly. Advances in analytical methods and decreased cost of measuring certain PFAS in water and other media broaden the ability of PWSs, regulatory and health agencies, academics, and nonprofits to identify water contamination. In June 2018, the Agency for Toxic Substances and Disease Registry (ATSDR) released a draft Toxicological Profile that derived minimal risk levels (MRLs), which are similar to RfDs, for intermediate duration exposure (15–364 days) of four PFAS routinely measured in NHANES [28]. The MRL values for PFOA (3 ng/kg/day) and PFOS (2 ng/kg/day) are 6.7 and 10 times lower than the RfDs EPA used to develop its 2016 HAs and similar to those developed by New Jersey, though they are based on different studies and endpoints. The release of this report became surrounded in controversy amidst suggestions that months earlier, EPA and other government officials sought to delay its release, citing concerns about public reaction [93], and demonstrates how political and economic factors can affect the timely development of health-protective guidelines.

In the absence of enforceable, nationwide water standards for PFAS, some states have developed more health-protective and scientifically sound guidelines. This may create or exacerbate public health disparities because not all states have the resources to develop guideline levels. The ability of states to develop their own guideline levels and standards provides diverse risk assessment approaches as models for other state and federal regulators, while a sufficiently protective, scientifically sound, and enforceable federal standard would provide more consistent protection.
