# Correction: Guideline levels for PFOA and PFOS in drinking water: the role of scientific uncertainty, risk assessment decisions, and social factors

**DOI:** 10.1038/s41370-019-0134-5

**Published:** 2019-03-29

**Authors:** Alissa Cordner, Vanessa Y. De La Rosa, Laurel A. Schaider, Ruthann A. Rudel, Lauren Richter, Phil Brown

**Affiliations:** 1 Department of Sociology, Whitman College, Walla Walla, WA USA; 2 Silent Spring Institute, Newton, MA USA; 3 Department of Sociology and Anthropology, Northeastern University, Boston, MA USA; 4 Department of Health Sciences, Northeastern University, Boston, MA USA

**Correction to:** **Journal of Exposure Science & Environmental Epidemiology**

10.1038/s41370-018-0099-9

published online: 8 January 2019

This paper was originally published under a standard licence. This has now been amended to a CC BY licence in the PDF and HTML.

