# Correction: Guideline levels for PFOA and PFOS in drinking water: the role of scientific uncertainty, risk assessment decisions, and social factors

**DOI:** 10.1038/s41370-020-0207-5

**Published:** 2020-02-06

**Authors:** Alissa Cordner, Vanessa Y. De La Rosa, Laurel A. Schaider, Ruthann A. Rudel, Lauren Richter, Phil Brown

**Affiliations:** 1 Department of Sociology, Whitman College, Walla Walla, WA USA; 2 Silent Spring Institute, Newton, MA USA; 3 Department of Sociology and Anthropology, Northeastern University, Boston, MA USA; 4 Department of Health Sciences, Northeastern University, Boston, MA USA

**Correction to: Journal of Exposure Science & Environmental Epidemiology**

10.1038/s41370-018-0099-9

In the original version of this article, we misinterpreted the timeline for Vermont’s PFOA and PFOS Health Advisories (HAs). We regret the error. We provide the corrected text here for reference.

**Page 6:**

In February 2016, in response to concerns about water contamination near an industrial facility, Vermont drafted a drinking water HA for PFOA of 20 ng/L that was finalized in March 2016 [1]. In May 2016, EPA finalized its lifetime HA of 70 ng/L for PFOA and PFOS individually or combined [2, 3]. Shortly after, Minnesota, building off the EPA’s 2016 risk assessments, developed state guideline levels of 35 ng/L PFOA and 27 ng/L PFOS that were lower than the EPA HAs [4, 5], and Vermont referenced the EPA’s risk assessment in revising its HA to 20 ng/L for PFOA and PFOS individually or combined [6].

**Page 10:**

Unlike some states where limited regulatory appetite and strong industry and political influence may slow progress on protecting public health by establishing PFAS water exposure limits, other states have developed scientifically sound PFAS guideline levels in response to discoveries of local contamination. For example, after the discovery of PFOA contamination in Hoosick Falls, New York, a resident in nearby North Bennington, Vermont raised concerns to local legislators. The state of Vermont reacted quickly, first creating a PFOA HA of 20 ng/L and then using that HA to develop a groundwater enforcement standard. Testing of private wells by Vermont’s Department of Environmental Conservation found PFOA concentrations well above the state’s HA, prompting the state to quickly provide bottled water and conduct additional water testing, soil sampling, and blood testing of local residents [7–9].
